# Molecular Dialog of *Ralstonia solanacearum* and Plant Hosts with Highlights on Type III Effectors

**DOI:** 10.3390/ijms26083686

**Published:** 2025-04-13

**Authors:** Xinyu Hu, Weiwei Cai, Laining Zhang, Zhujun Zhu, Thomas W. Okita, Li Tian

**Affiliations:** 1 Collaborative Innovation Center for Efficient and Green Production of Agriculture in Mountainous Areas of Zhejiang Province, College of Horticulture Science, Zhejiang A&F University, Hangzhou 311300, China; 13777356943@163.com (X.H.); caivivi@zafu.edu.cn (W.C.); laining.zhang@zafu.edu.cn (L.Z.); zhuzj@zafu.edu.cn (Z.Z.); 2 Key Laboratory of Quality and Safety Control for Subtropical Fruit and Vegetable, Ministry of Agriculture and Rural Affairs, Zhejiang A&F University, Hangzhou 311300, China; 3 Institute of Biological Chemistry, Washington State University, Pullman, WA 99164, USA

**Keywords:** *Ralstonia solanacearum*, type III effectors, pathogenesis, plant immunity

## Abstract

*Ralstonia solanacearum* is a highly destructive soil-borne bacterium that causes bacterial wilt disease in more than 310 plant species worldwide. The pathogenicity of the bacteria is closely associated with type III effectors (T3Es), a class of virulence factors that are delivered to host plant cells by the type III secretion system. In spite of the complex evolutionary history and genetic diversity of the *R. solanacearum* species complex (RSSC), more than 100 different T3Es have been identified from the genomes of various strains. Based on the available functional studies, certain T3Es interact with host plant proteins and suppress host cell immunity, whereas other T3Es are recognized by the host plant to trigger specific resistance mechanisms. This review summarizes the mechanisms by which T3Es interfere with plant immune responses and the activation of the plant defense system upon T3E recognition. This in-depth review of the molecular interactions between *R. solanacearum* and its host plants offers insights into the complexity of plant–pathogen interactions and provides a scientific rationale and theoretical support for the future breeding of resistant crops.

## 1. Introduction

The *Ralstonia solanacearum* species complex (RSSC) is ranked as the second most damaging bacterial phytopathogen [1]. These soil-borne bacteria are widely distributed in tropical, subtropical, and temperate regions worldwide [2,3], and cause the widespread disease known as bacterial wilt. They can infect over 310 plant species belonging to 42 families, including *Solanaceae* crops such as eggplant (*Solanum melongena*), tomato (*Solanum lycopersicum*), pepper (*Capsicum annuum*), and potato (*Solanum tuberosum*), *Leguminosae* crops, and dicotyledonous woody plants like mulberry, eucalyptus, and *Casuarina equisetifolia*, along with monocotyledonous plants like banana and ginger [1,4,5,6]. Due to its remarkable adaptability and potent destructive capabilities in diverse environments and multiple crop species [7], *R. solanacearum* poses a severe threat to global agricultural production, with crop yield losses ranging from 20% to 100% [1]. To ensure global food security and the sustainability of agricultural production, the research on bacterial wilt caused by *R. solanacearum* has received extensive attention over the years.

In response to bacterial pathogen invasion, plants activate the innate immune system to resist infection. During the long-term struggle against pathogens, plants have developed a two-tiered immune system. In the initial layer of defense, plants recognize pathogen-associated molecular patterns (PAMPs) via pattern recognition receptors (PRRs) located on the cell membrane and cytoplasm, a process referred to as PAMP-triggered immunity (PTI) [8]. The second layer of defense is effector-triggered immunity (ETI), which relies on the recognition of pathogen-encoded virulence factors by intracellular receptors, mainly nucleotide-binding leucine-rich repeat (NLR) receptors, and is thus sometimes referred as NLR-triggered immunity (NTI) [9]. ETI is typically characterized by a local programmed cell death response, the hypersensitive response (HR). This serves as a self-protective mechanism to impede the spread of pathogens to other regions of the infected plants and ultimately suppress pathogen growth within the host plants [10,11]. These two immune mechanisms cooperate to form a robust defense system, allowing plants to effectively fight a diverse range of pathogen threats. Nevertheless, pathogens are constantly evolving new strategies to evade or suppress plant immune responses, resulting in a perpetual tug of war of attack and defense between pathogens and host plants. The accumulating evidence indicates that pathogens not only suppress immune responses directly but also affect numerous indirect physiological and biochemical pathways in plants, such as modulation of protein metabolism, gene expression, redox metabolism, and hormonal signaling [12].

The goal of this review is to describe the pathogenic characteristics and virulence factors of *R. solanacearum*, as well as how the type III effector proteins (T3Es) of *R. solanacearum* augment bacterial pathogenicity by disrupting plant immune and metabolic pathways. Additionally, it highlights the mechanism by which plants identify T3Es via NLR receptors and subsequently activate immune responses.

## 2. The Complex Evolutionary History of *R. solanacearum*

*R. solanacearum,* formerly known as *Pseudomonas solanacearum*, comprises a heterogeneous species. It was initially classified into six biovars based on biochemical properties [13,14,15], and subsequently further subdivided into two divisions [16,17]. Division 1 is primarily isolated in Asia and consists of biovars 3, 4, and 5. In contrast, division 2 is composed of biovars 1, 2, and N2, which are mainly isolated in the Americas. According to the study conducted by Fegan and Prior in 2005 [18], *R. solanacearum* was renamed as the *R. solanacearum* species complex (RSSC), which comprises at least four genetic groups or phylotypes, each of which is closely associated with its respective geographical origins. Phylotype I is predominantly present in Asia and includes biological variants 3, 4, and 5. Phylotype II includes the biological variants 1, 2, and 2T from North America and Asia [19]. Phylotype III is mainly found in Africa and encompasses the strains from biological variants 1 and 2T. The evolutionary phylotype IV contains strains from Indonesia [20].

A multilocus sequence analysis (MLSA) was carried out on a global collection of *R. solanacearum* to explore the genetic imprints [21]. The study delineated a distinct evolutionary complex and characterized eight clades possessing distinct evolutionary dynamics. Based on the analysis of the identified 21 recombination events, phylotype IV was proposed to be a primary gene donor for the majority of the recombination events. Although continuous diversification was predominantly detected within phylotype I, IIA, and III, other factors, like adaptation to a specific host or the intense trading of infected crops, are likely to have influenced this diversification. The question of whether the lineages of *R. solanacearum* will ultimately evolve into distinct species remains unanswered. Recently, a comprehensive metagenomic analysis based on 100 representative RSSC genomes revealed that the *R. solanacearum* genome contains 4940 genes on average, among which 3262 genes are present in the core genome and 13,128 genes in the extensive flexible genome [22]. The study also found that a third sub-clade of phylotype II is the most diverse phylotype, and proposed that phylotype II may represent the ancestral group of the RSSC.

Along with a better understanding of the RSSC, a polyphasic taxonomic revision was further proposed to emend the description of the RSSC [20,23]. The study reclassified the RSSC into three genospecies, relying on the phylogenetic analysis of 16S-23S rRNA (ribosomal RNA) internal transcribed spacer (ITS) region gene sequences. One of the genospecies is composed solely of phylotype II strains. The second genospecies, namely *Ralstonia syzygii*, comprises the strains from phylotype IV, which are mainly identified in Indonesia. The third genospecies includes the strains belonging to phylotypes I and III, which were emended into *Ralstonia pseudosolanacearum*.

Although numerous studies have been conducted to classify the RSSC based on biochemical properties, host range at the plant species level, geographic distribution, and even phylogenetic relationships, ambiguity and conflicting viewpoints remain prevalent in the classification of the RSSC. The principal factor contributing to this may be the adaptive genetic diversity and the complex evolutionary dynamics during the long-term interaction between the RSSC and its extensive array of host plants. This host–pathogen interaction not only leads to genetic alterations within the RSSC but also influences its virulence mechanisms. As a consequence, it significantly augments the pathogenicity and aggressiveness of the RSSC during the host–pathogen association. In this review, to keep consistency and simplicity, the bacteria encompassed by the RSSC are uniformly designated as *R. solanacearum*, despite the acknowledged taxonomic intricacies and ongoing debates within the scientific community regarding their precise classification.

## 3. Main Virulence Factors of *R. solanacearum*

Given its wide host range and significant influence on global crop production, *R. solanacearum* has emerged as a prominent and extensively studied model in the field of plant–bacteria pathogen interaction. This pathogen is armed with a broad set of virulence factors, endowing it with the ability to inflict damage upon host plant cells and intricately regulate plant immune mechanisms. The key virulence elements comprise extracellular polysaccharides (EPSs), motility, type III secretion systems (T3SSs), bacteriophages, and cell wall-degrading enzymes. Each of these elements play an essential role in the establishment of infection and colonization of the pathogen in host plant cells [24].

EPS is among the major groups of secondary metabolites that are produced by a wide variety of microbes. Through the construction of an extracellular biomatrix, EPSs protect microbes against adverse environmental conditions, such as extreme temperature, dehydration, pH fluctuations, osmotic stress, and toxic substances [25]. In the context of infection, the secreted EPS fulfills multiple functions in relation to the virulence of *R. solanacearum*. EPS promotes the formation of bacterial biofilms [26] and facilitates the adhesion of bacteria to the root of host plants [27]. The abundant EPS produced by *R. solanacearum* physically blocks the xylem vessels of the infected plant, directly causing wilting due to the occlusion of water transport [28,29]. Meanwhile, EPSs cloak bacterial surface characteristics as well, enabling *R. solanacearum* to evade the detection by the plant immune system [30]. Mutants of *R. solanacearum* with diminished EPS production exhibited a substantially decreased level of virulence [31], providing direct evidence that the EPSs are crucial virulence factors.

Motility plays a significant role in determining the virulence of *R. solanacearum*. The polar flagella are responsible for the swimming motility of *R. solanacearum*. Mutations in flagellin biosynthesis-related genes, such as those encoding the flagellar filament subunit fliC and the flagellar motor switch protein fliM, led to a complete lack of motility in *R. solanacearum*, whether on agar plates, in liquid broth, or in tomato plants [32]. The rotation of flagellar in *R. solanacearum* is regulated by core chemotaxis signal transduction proteins like CheW, CheA, and CheY. The absence of either CheA or CheW abolishes the chemotaxis and virulence *R. solanacearum* [33]. The twitching motility of *R. solanacearum*, which is a flagellum-independent movement on solid surfaces, is usually governed by self-produced type IV pili (TFP). It has been demonstrated that the deletion of the *pili* gene had no effect on normal swimming and chemotaxis but resulted in a reduced twitching motility, abnormal formation of bacterial biofilm, and lower levels of root attachment [34]. These studies imply that both swimming and twitching motility are essential for the effective invasion carried out by *R. solanacearum* in the host plants.

Bacteriophages are intracellular parasites that specifically target bacterial cells. Some phages conduct a lysogenic life cycle, in which their genetic material integrates into the host bacteria chromosome, forming a prophage. The transfer of auxiliary genes from temperate prophage to bacteria can drive bacterial evolution, which may endow the bacteria with various advantageous traits such as antibiotic resistance, competitiveness, and virulence [35,36,37,38,39,40]. It has been established that the competitiveness and virulence of *R. solanacearum* are significantly influenced by its diverse accessory genome from temperate prophages [40]. For instance, temperate phages belonging to two distinct clades, labeled as RSS-type and RSM-type, can exert differential impacts on the virulence of the host bacterium by modulating the expression of virulence factors. The RSS-RSM intermediate φRS551 can also increase the competitiveness of the host bacterium through enhancing metabolic versatility [41,42]. A comprehensive study on 120 *Ralstonia* spp. genomes uncovered a large number of characterized prophages as well as novel ones. It was further proposed that the auxiliary genes originating from those prophages potentially play crucial roles in determining host virulence, cellular metabolism, environmental stress tolerance, and antibiotic resistance [43]. Similarly, another study conducted an extensive analysis of the putative prophage-encoded auxiliary genes in 192 *R. solanacearum* draft genome assemblies. The investigation identified 313 intact prophages, which were grouped into 10 genetically distinct clusters. This finding underscores the significant contribution of prophage diversity to the evolution and genetic diversity of the RSSC [40].

To break through the physical barrier presented by cell wall defenses, *R. solanacearum* frequently secretes cell wall-degrading enzymes to facilitate infection [44,45,46]. These hydrolytic enzymes include cellulase, cutinase, esterase, pectinase, invertase, xylanase, and xyloglucanase [47]. However, when *R. solanacearum* secrete cell wall-degrading enzymes, plants are capable of detecting the status of cell wall fragments that have been broken down by pathogens [48,49,50]. For instance, fibrous dextrins from cellulose and oligogalacturonic acid in pectinate can be sensed by the plant’s cell wall integrity (CWI) system. Upon detection, this system triggers a defense response, which is an essential part of the plant’s immune mechanism against pathogen invasion. This interaction between the pathogen’s offensive strategy and the plant’s defensive sensing and response is thus one of the crucial aspects of the complex relationship between *R. solanacearum* and its host plants.

## 4. Type III Effectors Secreted by Type III Secretion

Among the main virulence elements, the type III secretion system (T3SS) stands out as the primary virulence determinant of *R. solanacearum*. The T3SS is encoded by the hrp (hypersensitive response and pathogenicity) gene cluster, which contains over 20 genes [51]. These genes mainly encode proteins that are required for the assembly of complex pili and the secretion of effector proteins. The pili constructed by the T3SS span across the inner and outer membranes of the bacterium, enabling the direct secretion of effector proteins into plant cells [52]. Functioning like a “molecular syringe”, the T3SS directly injects type III effector proteins (T3Es) into host cells, which, therefore, are also referred to as *Ralstonia* Injected Proteins (Rips). Once translocated into the host cells, T3Es interfere with the host’s defense system and modulate the host’s metabolism, ultimately playing a role in determining the virulence of *R. solanacearum* [53].

T3Es are described as a large, diverse, and robust arsenal of *R. solanacearum* [53]. Based on extensive studies using either homology searches, genetic screening, or gene expression analysis, more than 110 T3Es have been identified from over 140 *R. solanacearum* strains [53,54,55], with an average number of 64 *T3E* genes and a varying range of 45–76 *T3E* genes carried by an individual strain. It is notable that the number of *T3E* genes present in *R. solanacearum* genomes is larger than that of other known bacterial pathogens like *Pseudomonas syringae* and *Xanthomonas campestris*, which possess an average of 31 and 23 *T3E* genes, respectively [53,56,57]. A previous pangenome analysis of *R. solanacearum* demonstrated that the meta-repertoire of T3Es could be categorized into 94 orthologous families based on phylogenetic relationships. The study renamed all the *T3E* genes, including the former designated effectors, such as *Pop* (Pathogenicity protein), *Avr* (Avirulence protein), *Brg* (Bacterial resistance gene), Rip (Ralstonia injected protein), *Hpx* (Hypersensitive response and pathogenicity protein), *Lrp* (Low-regulated protein), etc., and proposed a unified nomenclature for *T3E* genes with the usage of the general term of *RipXYZ*, wherein the gene (*X*) is indicated by alphabetic characters, paralogous genes (*Y*) in numerical characters, and the name of the strain (*Z*) in subscript if provided [58]. The nomenclature was thereafter widely used in naming T3Es [54]. Subsequent mining of the *R. solanacearum* genome yielded an additional 16 [58] and 10 [55] hypothetical *T3E* candidates, which further supplemented the T3E repertoire of *R. solanacearum*. On the other hand, each strain usually carries multiple genes belonging to these paralog T3E families, which may contribute to the large size of the *R. solanacearum* pan-effectome. Among these T3E families, over 71 of them have been experimentally proven to be translocated and secreted through the T3SS [58,59]. Such experimental approaches encompass large-scale omics-based screening, including transcriptomics, interactomics, and metabolomics, as well as specific studies on their localization, virulence, and biochemical activities [54].

Although several studies on the *R. solanacearum* genomes attempted to identify the core effectors among the RSSC strains, the results were inconclusive. For instance, only eight T3Es were found to be highly conserved in 84 *R. solanacearum* strains [55]. When the scope was narrowed down to 11 strains, the number of core T3Es increased to 22 [58]. When considering the host-specific T3E repertoires, it was reported that only eight and seven T3Es were shared by the pathogenic strains of tomato and eggplant, respectively [60]. In the case of the strains isolated from bananas, 44 strictly defined T3Es could be identified from 9 strains, with only 27 from 15 strains [55]. All these studies reveal that the T3E repertoires of *R. solanacearum* display significant diversity in effectome size and specificity within different strains and across various host ranges. Future studies that involve in-depth mining of the core or unique T3Es carried by various strains may provide more insights into the evolution and host-adaptive mechanism of *R. solanacearum*.

## 5. Basic Characteristics of T3Es

The identification of T3Es is usually based on sequence homology with previously described effector genes or on functional analysis. The main sequence feature of T3Es is the presence of a specific 25-nucleotide cis-element (TTCGn16TTCG), which is referred as the *hrpII* box, or the plant-inducible promoter (PIP) box motifs located in the transcription promoter region [61]. The element is required for hrpB-dependent activation, where hrpB is the key operon that regulates the expression of T3SS biosynthetic genes and pathogenicity-related genes in *R. solanacearum* [62]. As a result, gene expression analysis on the strains carrying mutations in *hrpB* has also been employed to identify potential T3Es [63].

With the exception of the sequence features in the promoter region, the N-terminal domains of T3E proteins usually possess a typical amino acid pattern. This pattern directs translocation of the T3Es into host plant cells via the T3SS [64]. However, not all the identified T3Es possess the N-terminal export signal sequence, and many of them exhibit a high degree of sequence diversity. It was suggested that start codon plurality should be considered during the prediction of T3Es based on sequence homology, as it may undermine the reliability and accuracy of predictions regarding translation initiation sites [54].

Another commonly used method to identify T3E genes is based on a calmodulin-dependent adenylate cyclase (CyaA’) reporter assay, which has facilitated the discovery of numerous T3Es [65,66,67]. Such an assay relies on the random insertion of a truncated form of CyaA’ into the bacterial genome [67]. If the insertion does not affect the functional translocation of the effector, the fusion protein formed with CyaA’ can lead to an increase in the accumulation of cyclic adenosyl monophosphate (cAMP) within the infected plant. Based on the levels of cAMP in plant cells, an effector can then be identified. Similar methods that take advantage of the translocation property of T3Es into plant cells via the T3SS have been widely employed to discover new T3Es and validate their functions. Strategies such as fusion expression with a tag followed by detection with immunoblotting [68] and the global assessment of T3E secretomes through proteomics approaches have contributed to the discovery of over 30 T3Es [69].

## 6. Molecular Dialog Between T3Es and Plants

Similarly to the situation in other pathogens, *R. solanacearum* T3Es collectively contribute to the pathogen’s fitness within the plant through various mechanisms [70]. While many have not been fully characterized, these mechanisms include interference with the plant basal defense responses, alteration of the plant metabolism, and avoidance of the specific recognition of other T3Es. Some *R. solanacearum* T3Es can also be recognized by specific plant genotypes, thereby inducing strong immune responses. In the subsequent sections, this review will summarize the current knowledge about the dual roles of T3Es, specifically their functions in promoting the virulence of the RSSC and in inducing host immune responses.

### 6.1. The Virulence Caused by T3Es

T3Es exert effects on multiple cellular processes, including gene expression, metabolism, signal transduction, and hormone biosynthesis [71]. Through these effects, they suppress plant immunity and contribute to pathogen virulence [72]. Such wide influence lies in the diverse localization and functions of T3Es within plant cells.

#### 6.1.1. Modulation of Host Plant Protein Metabolism

Many T3Es have been reported to interfere with the protein metabolism of host plants. Some of them, like RipAW and RipAR, possess a novel E3 ubiquitin ligase (NEL) structural domain. When compared to other effectors identified from animal- or plant-pathogenic bacteria, they imitate the function of the homologous E6AP C-terminus (HECT)- and really interesting new gene (RING)-type E3 ubiquitin ligases [73]. These ligases are involved in the ubiquitination process, a highly conserved post-translational modification mechanism that is unique to eukaryotes and is used to regulate protein function in plant cells. It has been found that RipAW and RipAR are located in the cytoplasm of the host plant cells and exhibit E3 ubiquitin ligase activity in vitro. The E3 ubiquitin ligase activities of RipAW and RipAR were proposed to be necessary for suppressing pattern-triggered immunity (PTI) [74]. In yet another study in *N. benthamiana*, researchers found that RipAW induces cell death in a manner that depends on its activity [75]. However, the E3 ligase activity of RipAW is not a necessary condition for triggering plant immune responses, suggesting a potential independence between effector-induced cell death and immune reactions. Another effector, RipV2, was reported to possess robust E3 ubiquitin ligase activity in vitro [76]. The transient expression of RipV2 has been observed to induce cell death in *Nicotiana benthamiana* and effectively suppresses flg22-induced plant innate immune responses through its enzymatic function. Further research is needed to fully understand the complex interplay between T3Es and their E3 ubiquitin ligase activities, as well as how these interactions may vary across different host plant species and under different environmental conditions.

RipG (former GALA) family effectors, which typically carry the F-box domain and leucine-rich repeats (LRRs), have been reported to affect plant disease resistance by interfering with the host’s ubiquitination pathway [77,78,79]. The F-box domain is required for the participation of RipG effectors in the formation of the Skp1-cullin 1-F-box (SCF) ubiquitin ligase complex through binding to Skp1 [61,79]. Based on yeast two-hybrid screening, chloroplastic proteins might be the targets of RipG effectors, particularly RipG2 and RipG7, and the targeting is dependent on their LRR domains or their N-terminal regions [77]. While these findings provide new insights into the mechanisms by which RipG effectors manipulate host plant cellular processes, further investigations are required to elucidate their target proteins.

Among the identified effectors that manipulate host protein metabolism, RipAC is the one that has been relatively thoroughly studied. RipAC is a leucine-rich repeat (LRR) motif-containing effector. It is reported that RipAC targets the plant U-box protein 4 (PUB4) and SUPPRESSOR OF G2 ALLELE OF skp1 (SGT1) to suppress PTI and ETI, respectively [80,81]. PUB4 is a crucial E3 ubiquitin ligase that positively regulates PTI through promoting the homeostasis of the central immune kinase, BOTRYTIS-INDUCED KINASE1 (BIK1) [82,83]. RipAC impacts the accumulation and phosphorylation of PUB4, which in turn results in the degradation of BIK1 and the failure of PTI [81]. SGT1 plays an essential role in regulating the accumulation and activation of NLR proteins during ETI [80]. Although RipAC is unable to affect the accumulation of SGT1, it inhibits the phosphorylation of SGT1 by impeding the interaction between SGT1 and MAP kinases in *Arabidopsis* and tomato plants. Such phosphorylation is required for the activation of immune responses mediated by the NLRs [80,84]. In tobacco, RipAC was found to interact with NbSGT1, which inhibits the formation of a molecular chaperone complex of NbSGT1 and NbRAR1 to suppress ETI [85]. The abovementioned studies imply that some T3Es may function as molecular chaperones to hijack E3 ubiquitin ligase and, in turn, interfere with the host’s protein metabolism. However, the specific proteins that are targeted by T3Es remain largely unidentified, which requires further investigation.

#### 6.1.2. Subversion of Host Plant Transcription

Several nucleus-localized T3Es have been reported to disrupt gene transcription in host plants. Such influence results in large-scale changes in gene expression, especially the expression of the genes related to disease resistance. A study from tobacco plants investigated the subcellular localization of eight T3Es, namely RipA1, RipAB, RipAD, RipAF1, RipAO, RipD, RipE1, and RipL, which carry a predicted nuclear localization signal (NLS) [86]. Among these effectors, only RipAB and RipAO were found to exclusively localize in the nucleus, while RipAF1 localized in both the nucleus and cytoplasm. The remaining effectors were detected in the cytosol, chloroplast, endoplasmic reticulum (ER), or vesicle-like structures. Although they have diverse subcellular localization, most of these effectors caused significant changes in the expression of genes related to PTI. While the influence on transcription by most of these effectors has not been thoroughly studied, it has been reported that RipAB physically interacted with TGACG SEQUENCE-SPECIFIC BINDING PROTEIN (TGA) transcription factors in the nucleus, thereby dampening TGA-mediated plant immune gene expression [87]. This interaction influences the recruitment of RNA polymerase II at the transcription start site of TGA regulons, thereby inhibiting the TGA-mediated activation of immune genes, including salicylic acid (SA) signaling-related genes and the target genes responsible for PTI-triggered reactive oxygen species (ROS) burst like RESPIRATORY BURST OXIDASE PROTEIN D (RBOHD) and F (RBOHF). In addition to its effect on TGAs, RipAB also significantly reduces the expression of PTI-related immune-responsive genes like FRK1, PR1, WRKY30, and WRKY70 in *Arabidopsis* and tomato plants [87]. RipAB has also been reported in the interaction between *R. solanacearum* and potato plants, where it reduces the gene expression of the calcium ion (Ca^2+^) sensor and transporter [88]. The disruption of the Ca^2+^ signaling pathway diminishes ROS generation and, in turn, suppresses the plant’s immune response.

The *Yersinia* outer protein J (YopJ) superfamily of acetyltransferases, which are found in both animal and plant pathogens [89], modulates host immune responses by acetylating key amino acid residues like serine, threonine, and/or lysine residues on host proteins. This acetylation process inhibits the enzymatic activity or ATP binding properties of the target proteins [90,91,92]. Once translocated into plant cells, their enzymatic activities as acetyltransferases are allosterically activated through association with the eukaryote-specific ligand inositol hexaphosphate (InsP6), which stabilizes the substrate recognition helix in the target protein binding site [93]. Among these acetyltransferases, RipP2 (formerly PopP2) is localized in the plant cell nucleus and has the ability to specifically bind to the host WRKY transcription factors like WRKY41, WRKY70, and WRKY33 [94]. By specifically acetylating the conserved lysine residue in WRKY transcription factors, RipP2 effectively inhibits the DNA binding activity of WRKY, and consequently prevents the activation of plant defense genes.

In addition to TGA and WRKY transcription factors, bHLH family transcription factors may also be the targets of T3Es. Another nucleus-localized effector, RipI, was reported to interact with the bHLH93 transcription factor in *N. benthamiana* to regulate defense responses [95,96]. When *bHLH93* was silenced, the ability of RipI to induce HR and the expression of a defense gene PDF1.2 was reduced [96], suggesting that RipI specifically targets bHLH93.

Except for interacting with host transcription factors, some T3Es function as transcription factors to impact gene transcription within host plant cells. Brg11 belongs to the superfamily of RipTAL [97] and functions as a transcription activator-like effector (TALE). In eggplant, nucleus-localized Brg11 exhibits a specific binding to promoters containing a matching effector-binding element (EBE). Unlike the preference of the *Xanthomonas* TAL effector (TALE) AvrBs3 for sequences with a 5′ thymine, Brg11 preferentially binds EBEs that possess a 5′ terminal guanine. Such direct binding may contribute to the virulence caused by Brg11 and other RipTALs through the transcriptional activation of host susceptibility genes, which offers new possibilities for the molecular breeding of plants resistant to *R. solanacearum*. On the other hand, a further study on Brg11 indicated that it exclusively targeted a 17 bp sequence, termed the arginine decarboxylase (ADC)-box, which is located upstream of ADC genes that are involved in polyamine biosynthesis [98]. The specific effect impeded the translation control of ADC, thereby elevating polyamine levels and triggering a defense reaction. Interestingly, the elevated levels of ADC and putrescine caused by Brg11 enhanced the resistance toward *P. syringae* in tomato but not *R. solanacearum*, suggesting that Brg11 confers a competitive advantage of *R. solanacearum* endued by Brg11 [98].

RipX (popA) belongs to the harpin-like protein family [99]. Members of this family are usually glycine-rich and heat-stable proteins that are secreted through the T3SS by plant-pathogenic bacteria [100]. Previous studies indicated that hairpins acted as elicitors to induce plant HR reaction and hormone signaling during pathogens or insect attacks [101,102], which may associate with altered mitochondrial function [103]. Although the subcellular localization of most harpin-like effectors after being translocated into plant cells remains largely unknown, RipX was reported to be located in the cytoplasm and plasma membrane. It interacted with mitochondrial ATP synthase F1 subunit α (ATPA) and also suppressed the transcription of the *atpA* gene. However, the specific mechanism by which RipX affects gene transcription requires further investigation.

#### 6.1.3. Manipulation of Plant Hormone Networks

During the infection process caused by *R. solanacearum*, plant hormones such as SA, JA, and ethylene play pivotal roles in coordinating the immune response. These hormones interact with one another, thereby forming an elaborate and intricate regulatory network to regulate plant growth, development, and the plant’s defense mechanisms. Among them, SA and JA are particularly vital in adjusting and modulating the plant’s responses to pathogen attacks [104,105]. Moreover, gibberellins (GAs), which mainly function to regulate plant growth and developmental processes, can also be utilized by pathogens to subvert host defense system [106].

RipN, identified from *R. solanacearum* strain GMI1000 [107], has been found to locate in the nucleus and the endoplasmic reticulum (ER) [107]. The effector contains a putative nudix hydrolase domain, in which nudix refers to a conserved group of organic pyrophosphate hydrolases that can hydrolyze a variety of nucleoside diphosphate derivatives, including NADH (Nicotinamide adenine dinucleotide), NAD+(Nicotinamide adenine dinucleotide), ADP-ribose (ADPr), phosphoinositol proteins, nucleoside triphosphates (NTPs) and deoxynucleoside triphosphates (dNTPs) [108,109]. When expressed in *Arabidopsis*, RipN was found to alter the NADH/NAD+ ratio in plant cells, and the alteration was dependent on its ADPr/NADH pyrophosphorylase activity. The impact of RipN on NADH/NAD+ homeostasis may contribute to the influence on plant hormone signal transduction, particularly leading to a notable increase in the levels of GAs and JAs [110].

RipAF1 has been recently reported to ADP-ribosylate the host fibrillin FBN1 (Fibrillin 1) [111]. FBN family protein are widely characterized as being involved in the biogenesis of chloroplast plastoglobules, response to different stress stimuli, and to JA and ABA signaling [112,113]. The ADP-ribosylation conducted by RipAF1 occurred on the conserved residues of FBN1, which contributes to the localization of FBN1 to the plasma membrane and leads to the suppression of JA signaling and induction of SA signaling [111].

RipAL, which contains a DEFECTIVE IN ANTHER DEHISCENCE1 (DAD1)-like lipase domain, localizes to chloroplasts and targets chloroplast lipids [114]. The effector causes a significant upsurge in the levels of JA and JA–isoleucine levels while simultaneously suppressing SA biosynthesis and signaling. Mutations in the DAD1-like lipase domain abolish its ability to induce JA and suppress SA, suggesting the key role of the lipase domain in RipAL-dependent infection. By intricately altering the JA and SA signaling pathways, RipAL creates a favorable environment for the pathogen, thereby facilitating its successful invasion and proliferation within the host plant.

Some other effectors have been reported to target hormone-responsive factors. RipE1 is a homolog of cysteine protease effector HopX1 from the *P. syringae*. The effector localizes in the nucleus and the cytoplasm and is found to interact with several jasmonate-ZIM-domain (JAZ) proteins, including JAZ4, JAZ9, and JAZ10, which are transcriptional repressors of the JA signaling pathway in plants [95]. Owing to the cysteine protease activity of RipE1, such interaction facilitates the degradation of JAZ proteins and induces the activation of JA-responsive genes. Moreover, RipE1 concurrently suppressed the SA signaling pathway by down-regulating the expression of the genes related to SA synthesis, thereby dampening the plant’s immune response [95,115]. In pepper plant, RipAK was found to target an ethylene-responsive factor ERF098. RipAK inhibited the transcriptional activity of ERF098 through impeding the homodimerization of ERF098. This inhibition further suppressed the transcriptional activation of the SA-dependent *PR1* gene, along with the dehydration tolerance-related *OSR1* (Osmotic Stress Resistance 1) and *OSM1* (osmotin-like protein 1) genes. As a consequence, both the plant immunity and dehydration tolerance were decreased, ultimately resulting in the occurrence of bacterial wilt in pepper [116].

The impacts on plant hormone signal transduction exerted by T3Es are quite complicated. Many aspects of T3E-mediated hormonal regulation still remain to be explored, including the identification of additional target proteins and the precise mechanisms by which they exert their effects on different hormonal pathways under different environmental and physiological conditions.

#### 6.1.4. Impact on Host Plant ROS Homeostasis, HR Response, and Beyond

Plant secondary metabolites are essential components of the plant’s defense system and play a direct and significant role in protecting plants against disease. However, pathogens have also evolved complex infection strategies over time to evade the plant defense systems. Among these strategies, the T3Es of plant pathogens specifically target and manipulate the host’s metabolic processes, which constitutes a crucial mechanism to facilitate the survival of the pathogen, the acquisition of nutrients, and the spread of infection throughout the plant [12,72].

During the interactions between plants and pathogens, reactive oxygen species (ROS) serve as second messengers to transduce diverse plant defense signals against both biotic and abiotic stresses. The T3Es secreted by *R. solanacearum* have the ability to manipulate ROS-mediated cellular signaling, thereby influencing plant immune response [117]. RipB and RipAA are among these effectors that hijack plant ROS homeostasis as a means to achieve immune evasion. When the GMI1000 strain carrying mutations of *ripB* was applied to infect *Arabidopsis* plants, it was observed that hypersensitive response (HR)-induced cell death was impaired [118]. Meanwhile, its expression reduced plant root growth and increased susceptibility to *R. solanacearum* infection through a partial suppression of ROS production [118]. While the detailed mechanism underlying the suppression of ROS burst caused by RipB needs further investigation, RipAA was reported to specifically target chloroplastic AtpB in *N. benthamiana* [119]. The expression of RipAA induced the accumulation of H2O2 and the degradation of genomic DNA within plant cells. These phenomena were accompanied by an HR response. Moreover, when *atpB* was silenced in *N. benthamiana*, the induction of HR caused by RipAA failed to occur, and the plant’s resistance to bacterial wilt was reduced.

Another effector that may interfere with plant ROS homeostasis is RipBT, a plasma membrane-localized effector recently identified using the cyaA reporter system [120]. Based on transcriptomic analyses, a mutation in *ripBT* attenuated the virulence of *R. solanacearum* in potato, which may be due to a failure in down-regulating the ROS production-related genes, including NADPH oxidases (respiratory burst oxidase homologues, RBOHs). Meanwhile, the expression of RipBT remarkably suppressed flg22-induced ROS burst and enhanced potato susceptibility to *R. solanacearum* [120]. This study supports a key role of RipBT in promoting *R. solanacearum* infection in potato by disturbing ROS homeostasis.

Other effectors, such as RipC1, RipAE, RipU, and RipW, were also reported to strongly suppress PTI-triggered ROS burst [121]. Among them, RipU locates in the nucleus, RipAE is found in the nucleus and on the plasma membrane, RipC1 locates on the PM and in the Golgi apparatus, and RipW is associated with the nucleus and the ER. It is worth mentioning that three of them, RipAE, RipU, and RipW, were found to induce cell death and influence mitogen-activated protein kinase (MAPK) cascades, while RipAE and RipW interfered with the SA and JA pathways, respectively [121]. The study suggests that the T3Es involved in disturbing ROS homeostasis are located in diverse subcellular locations.

In tobacco plants, RipAK is found in peroxisomes [122] and promotes the virulence of *R. solanacearum* by reducing the host’s HR response. This is achieved through its interaction with host catalases (CATs) that are critical enzymes for scavenging ROS and maintaining redox homeostasis. The inhibition of CAT activity disrupts ROS signaling, thereby suppressing the plant’s early immune response to infection [122]. While knockout of *RipAK* in *R. solanacearum* strain GMI1000 resulted in an obvious HR response and a prompt accumulation of ROS, its overexpression inhibited the transcription of many immunity-associated genes in tobacco plants [122]. Moreover, RipAK was reported to interact with pyruvate decarboxylases (PDCs), which are involved in plant tolerance to bacterial wilt [123]. The interaction inhibits PDC oligomerization and, in turn, enzymatic activity in *Arabidopsis*, which likely contributes to the effective infection by *R. solanacearum*.

Other T3Es that potentially have a function in disrupting plant metabolism are RipA5, RipI and RipAY. It has been reported that RipA5 can reduce nitrate reductase activity, influence nitrogen assimilation, and thereby facilitate *R. solanacearum* infection [124]. This may benefit from the function of RipA5 as an inhibitor of the target of rapamycin (TOR), a conserved serine/threonine protein kinase in eukaryotes. TOR precisely regulates cell growth and metabolic homeostasis through modulating processes like transcription, translation, and autophagy [125,126]. The TOR signaling pathway is also the target of the T3E AWR5 [124]. In yeasts, AWR5 might carry out its function by directly or indirectly inhibiting the TOR pathway upstream of PP2A phosphatase.

*R. solanacearum* is able to utilize gamma-aminobutyric acid (GABA) as a nutrient for reproduction. The effector protein RipI interacts with plant glutamate decarboxylases (GADs) and catalyzes the biosynthesis of GABA, thus helping *R. solanacearum* to obtain nutrients more efficiently from tomato plants [127]. RipAY undermines plant immunity by specifically degrading glutathione (GSH) within the plant cells. This degradation is achieved by its gamma-glutamyl cyclotransferase (GGCT) activity, which is activated by plant cytosolic h-type thioredoxins (TRX-h) [128,129]. The subsequent GSH depletion attenuates the plant’s PTI response, reducing both the production of ROS and the expression of immunity-associated genes. Notably, RipAY remains inactive within bacterial cells, demonstrating a precision-targeting safety mechanism that prevents self-intoxication. This mechanism endows *R. solanacearum* with an effective strategy to evade the host plant’s immune defenses [128,129].

Protein phosphatase I (PP1), also called the type one protein phosphatase (TOPP), is an essential eukaryotic serine/threonine phosphatase required for many cellular processes, including transcription, cell division, signaling, and metabolism [130]. The role of TOPP proteins in plant defense is demonstrated in *Arabidopsis* through the autoimmune phenotype of the *topp4-1* mutant and the enhanced pathogen resistance observed in the septuple mutant of *topp1/4/5/6/7/8/9* [131]. This regulatory function is likely mediated by the physical interaction of TOPP proteins with MAPKs, thereby influencing the MAPK-mediated downstream defense pathway. In potato, PP1 protein StTOPP6 was found to negatively regulate potato bacterial wilt resistance by modulating MAPK signaling [132]. Knockdown or inactivation of StTOPP6 enhances resistance by activating immune responses, including the MAPK3-mediated pathway and reactive oxygen species (ROS) accumulation, while StTOPP6 suppresses these defenses, highlighting its role in promoting disease susceptibility. Another study reported that RipAS promoted bacterial wilt by interacting with and reducing the nucleolar accumulation of StTOPP6, thereby suppressing plant immunity and enhancing pathogen virulence in potato [133]. This interaction is critical for RipAS-mediated disease progression, as evidenced by the impaired resistance in RipAS-expressing plants and the widespread association between RipAS and other PP1 family members.

**Figure 1:**
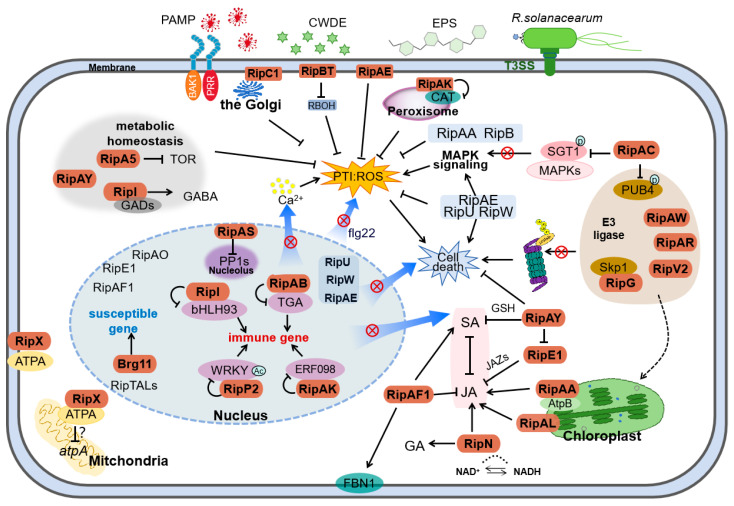
Mechanisms of *R. solanacearum* T3E-mediated suppression of plant immunity. For detailed information, please refer to the main text. ⨂ represents that the corresponding pathways is inhibited, and ? indicates that the underlying mechanism is unknown. EPS, exopolysaccharide; CWDEs, cell wall-degrading enzymes; GSH, glutathione; FBN1, Fibrillin 1; PUB4, plant U-box protein 4; ATPA, ATP synthase F1 subunit α; RBOHs, respiratory burst oxidase homologs; CAT, catalase; TOR, the target of rapamycin; GABA, γ-aminobutyric acid; GADs, glutamate decarboxylases.

To sum up, the mechanisms of *R. solanacearum* T3E-mediated suppression of plant immunity are summarized in Figure 1. During the infection process, *R. solanacearum* produce virulence factors, including EPS, cell wall-degrading enzymes, and T3SS-delivered effectors. After being injected into the host cell, T3Es are translocated to different subcellular locations. Nucleus-localized T3Es, like RipI, RipAK, RipAB, and RipP2, physically interact with host transcription factors, including bHLH93, ERF098, TGA, and WRKY family members, to suppress the expression of immune genes. Brg11 and other RipTALs may bind to the host DNA and transcriptionally activate the host susceptible genes. RipAS promotes bacterial wilt by interacting with and reducing the nucleolar accumulation of PP1-like proteins (e.g., StTOPP6). The detailed function of some other nucleus-localized RipAO, RipE1, RipAF1, RipU, RipW, and RipAE are largely unknown, although RipU, RipW, and RipAE may function in suppressing the host ROS burst and activating MAPK signaling. The manipulation of host transcription by nucleus-localized T3Es may cause the disturbance of SA and JA biosynthesis and signaling, Ca^2+^ signaling, and induction of cell death and ROS burst. RipAY and RipE1 suppress SA and JA signaling transduction through degrading glutathione (GSH) or inhibiting JAZs, respectively. The cytoplasm-localized RipAF1 ADP-ribosylates the host fibrillin FBN1 (Fibrillin 1), which contributes to the localization of FBN1 to the plasma membrane and leads to the suppression of JA signaling and induction of SA signaling. RipN alters the NADH/NAD+ ratio, which promotes JA signaling. Both RipAA and RipAL target chloroplasts and induce JA production, in which RipAA specifically targets chloroplastic AtpB. RipAW, RipAR, and RipV2 act as E3 ligases to modulate the host plant protein metabolism. RipAC impacts the accumulation and phosphorylation of the plant U-box protein 4 (PUB4). RipAC is able to interact with and affect the accumulation and phosphorylation of SGT1, which impedes the interaction between SGT1 and MAP kinases and thus ETI. RipX, which locates to the cytoplasm and plasma membrane, interacts with mitochondrial ATP synthase F1 subunit α (ATPA) and suppresses the transcription of the atpA gene. The plasma membrane-localized RipBT influences the expression of many several ROS production-related genes like respiratory burst oxidase homologues (RBOHs) to suppress ROS burst. Other plasma membrane-localized T3Es include RipC1 and RipAE, whereas RipC1 is also located on the ER. Both of them strongly suppress PTI-triggered ROS burst. The peroxisome-localized RipAK interacts with host catalases (CATs) to disrupt ROS signaling. The cytoplasm-localized RipA5, RipAY, and RipI alter the host cell metabolic homeostasis. RipA5 functions as an inhibitor to the target of rapamycin (TOR) while RipI disrupts γ-aminobutyric ccid (GABA) metabolism via glutamate decarboxylases (GADs).

Collectively, T3Es act as critical virulence determinants by manipulating diverse host cellular processes to subvert plant immunity and facilitate infection. As outlined in Figure 1, these effectors target multiple plant defense pathways, including suppression of PTI and ETI, modulation of phytohormone signaling networks, perturbation of host primary metabolism, and dysregulation of transcriptional programs. While significant progress has been made in identifying T3E functions, the molecular mechanisms underlying their interactions with host proteins remain poorly understood in most cases. This knowledge gap highlights the need for integrative approaches combining structural biology, system biology, and reverse genetics to dissect these interactions and decipher their roles in pathogenesis.

### 6.2. Host Resistance Mediated by Recognition of T3Es

#### 6.2.1. Avirulence Effectors and Their Roles in HR and ETI-Mediated Host Resistance

The concept of avirulence (Avr) effectors was initially proposed in the 1940s by Harold Henry Flor. *Avr* genes are usually pathogen genes that encode a protein that is conditionally recognized by plants carrying the corresponding resistance (*R*) gene. The proposal was based on the gene-for-gene hypothesis that was derived from his study on the interaction between flax and the rust pathogen, *Melampsora linithe* [134]. According to the hypothesis, a plant carrying a resistance (*R*) gene has the ability to resist the pathogens that possess complementary *Avr* genes. In other words, when the specific *R* gene in the plant is paired with the corresponding *Avr* gene in the pathogen, it induces resistance in the plant. However, it is still possible that even when a plant harbors an *R* gene, the pathogen that possesses the paired avirulence gene may still cause disease [135].

Upon the recognition of Avr effectors, a series of defense mechanisms are activated within the plant. These include the activation of diverse signaling pathways, such as the MAPK pathway, Ca^2+^-dependent signaling, and ROS signaling, and the induction of defense-related genes. This may lead to the production of antimicrobial compounds, reinforcement of the cell wall, and the initiation of HR response. Among them, HR response is a rapid and localized cell death around the site of infection to limit the spread of the pathogen.

**Figure 2:**
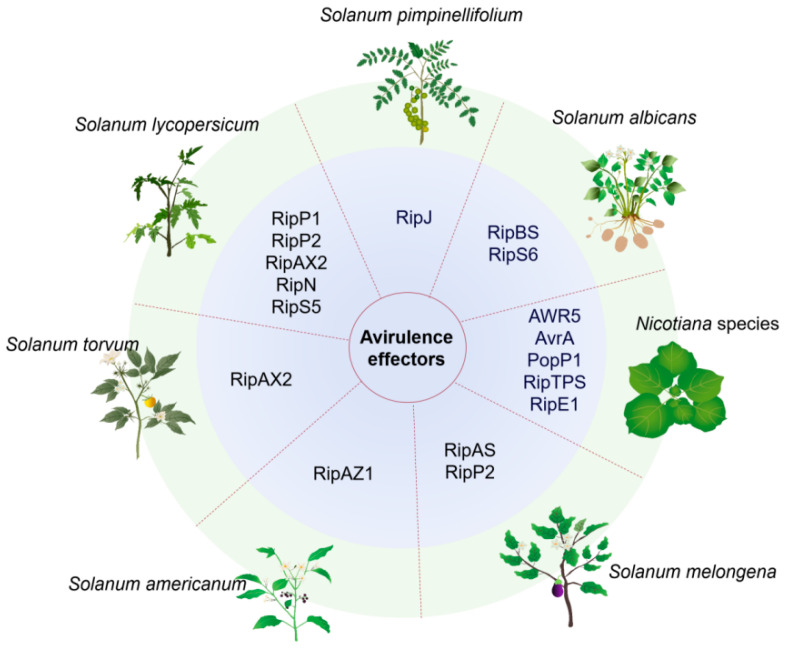
Diverse host range and specific recognition of *R. solanacearum* T3Es by different Solanaceae and Nicotiana species. These T3Es act as avirulence effectors and may be utilized to attenuate *R. solanacearum* virulence and enhance the host resistance. Rip, Ralstonia injected protein; Avr, avirulence protein.

Recently, a growing body of research focusing on the avirulent effectors of *R. solanacearum* has emerged in Solanum species (Figure 2). The first *R. solanacearum* avirulence effector is RipJ, which was reported to trigger bacterial resistance in tomato *Solanum pimpinellifolium* LA2093 [136]. RipJ belongs to the YopJ family of acetyltransferases. However, the conserved amino acid residues in the acetyltransferase catalytic triad are not required for effector-triggered immunity. The effector protein RipBJ, which is present in several *R. solanacearum* strains with low to moderate virulence, has been reported to be involved in the ROS burst [137]. The involvement is achieved through direct interaction with NADPH oxidase SlWfi1, thereby triggering plant cell death and the accumulation of ROS in tomato. RipAZ1 from *R. solanacearum* strain Pe_26 has been identified as a crucial avirulence determinant in *Solanum americanum* [138]. The effector localizes to both the nucleus and the cytoplasm, and possesses the ability to trigger cell death in the resistant cultivar SP2273 but not in the susceptible cultivar SP2275. Although the detailed mechanism of how RipAZ1 boosts plant immunity in the resistant strain is still unknown, the C-terminus of RipAZ1 is proposed to be required for its capacity to induce cell death.

Several avirulent effectors have also been identified from *Nicotiana* species (Figure 2). An analysis of the virulence of the AWR (alanine–tryptophan–arginine triad) effector family in *N. benthamiana* revealed that while AWR2 is the major contributor to virulence, AWR4 and AWR5 restrict the growth of *P. syringae* pv. tomato DC3000 [139]. In the case of AWR5, its overexpression in tobacco species triggers a rapid increase in the expression of harpin-induced 1 (HIN1), which is an HR-specific marker. As a result, cell death and H_2_O_2_ accumulation occur, both of which serve as indicators of an HR-like immune response [139,140]. The *avr* genes *avrA* and *popP1* (*ripAA* and *ripP1*, respectively, in unified nomenclature) are both responsible for the avirulence of the strain GMI1000 in different Nicotiana species by contributing to the elicitation of HR [141,142]. AvrA is the major determinant recognized by *N. tabacum* and *N. benthamiana*, while PopP1 appears to be the major HR elicitor in *N. glutinosa* [142]. A comparative study was conducted on a virulent strain OE1-1 and four HR-eliciting strains, and it was found that popP1 was absent in OE1-1 [143]. When popP1 was introduced into the virulent strain OE1-1, the resulting transconjugant strain exhibited a significant reduction in its virulence.

The diversity of *avrA* genes was comprehensively analyzed in the *R. solanacearum* population [140]. The study identified the wild-type *avrA* gene, which was capable of eliciting an HR reaction in tobacco. This reaction was characterized by necrotic lesions occurring at 16 to 24 h post-inoculation [140]. An 18-amino acid region in AvrA was proposed to be responsible for the HR elicitation in *N. benthamiana* [142]. Further analyses on *avrA* alleles revealed the presence of a DNA sequence containing two putative miniature inverted-repeat transposable elements (MITEs) [140] and a hypervariable region [142]. *avrA* appears to be the target of various DNA insertions or mobile elements, which probably allow *R. solanacearum* to evade recognition and defense responses in plants [142]. While these results indicate that AWR5, AvrA, and PopP1 could function as avirulence determinants of certain *R. solanacearum* strains to various *Nicotiana* species, other unidentified factors may be necessary for full virulence or HR elicitation.

Another respective effector secreted by *R. solanacearum* during tobacco infection is RipTPS. RipTPS is named after its trehalose-6-phosphate (T6P) synthase (TPS) activity. T6P is a key signaling molecule that regulates carbon assimilation and sugar metabolism in plants. Owing to its TPS activity, RipTPS promotes the production of T6P and elicits a HR-like response in tobacco [64]. Among the identified RipTPS, the effector RipTPSG, which was identified from the GMI1000 strain, is reported to selectively induce cell death in different tobacco varieties [144], suggesting that a specific recognition mechanism exists between the host plant and T3Es. While the expression of RipTPSG reduced the virulence of a virulent strain CQPS-1, its expression also induced the activation of HR-related genes in *N. tabacum*. A further investigation into RipTPSG and its homolog, RipTPSC, in CQPS-1 revealed that although both of them could suppress flg22-induced ROS burst in *N. benthamaina*, three core amino acid polymorphisms were jointly required for the recognition of RipTPSG in *N. tabacum*. In the case of RipTPSG, the difference in specific amino acids in the T3E indicates a divergence in evolution during interaction between host plants and various bacterial strains.

Along with the development of genomic techniques, the identification of avirulent effectors could also be conducted through a comparative analysis between resistant and susceptible genotypes or between avirulent strains and virulent strains to a specific plant species. Five core effectors, namely RipE1, RipH2, RipV1, RipV2, and RipX, which are secreted by *R*. *solanacearum* race 3 biovar 2 (R3bv2), were reported to trigger substantially stronger cell death in the resistant eggplant genotype MM853 than in the susceptible genotype MM738 [145]. It was thus speculated that the eggplant genotype MM853 may harbor *R* genes that recognize one or more of these core effectors. A study compared the virulence of *R. solanacearum* strains HA4-1 and HZAU091 in the wild potato *Solanum albicans* 28-1 (ALB28-1) [146]. It was found that HZAU091, but not HA4-1, was virulent to the wild potato. Several distinct T3Es between HZAU091 and HA4-1A were then identified by a comparative genomic analysis. Among them, RipBS and RipS6 from HA4-1 were found to significantly reduce the virulence of HZAU091 to wild potato and induce a hypersensitive response (HR) in leaf tissue [146]. This study further demonstrates that analysis on the T3Es present in avirulent strains can offer a useful approach to identify core avirulent effectors for plants that lack well-known resistance resources.

A study explored the T3E repertoire groups among *Arabidopsis thaliana*, bacterial wilt-resistant tomato Hawaii7996, and eggplant cultivars Dingras multiple Purple and AG91-25. It was proposed that virulence and avirulence phenotypes could not be explained by specific T3E repertories, but rather by individual T3E genes present in virulent and avirulent strains in relation to the specific cultivars [147]. In the study, RipA5_2 was identified as a virulence-associated T3E of the *R. solanacearum* strain PTO1391 during infection to eggplant cultivar Dingras multiple Purple, and RipU might be responsible for the virulence of the *R. solanacearum* strain PTO3560 in AG91-25 and tomato Hawaii7996. Among the identified avirulence effectors, RipAS potentially initiates an immune response in Dingras multiple Purple that could be integral to preventing *R. solanacearum* infections. In the case of AG91-25, RipAX2 and RipP2 were specifically recognized through a major resistance locus EBWR9 (ERs1) [147,148,149]. RipAX2, formerly named Rip36, which was initially identified in the *R. solanacearum* RS1002 strain, was previously reported to induce ETI in the wild eggplant *Solanum torvum*. The induction relies on its putative zinc (Zn)-binding motif (HELIH), as a mutation of E to A in the 149th amino acid in RipAX2 abolishes the structure of the zinc-binding motif and leads to the abrogation of the HR response, thereby allowing unrestricted bacterial proliferation within *S. torvum* [150]. However, the resistance of AG91-25 does not rely on this motif, suggesting a different mechanism may exist for perceiving RipAX2 [144]. The resistance of the Hawaii7996 cultivar against bacterial wilt is governed by multiple quantitative trait loci (QTLs) [151], and the avirulence phenotype observed in this cultivar is associated with the genes *ripP1*, *ripP2*, *ripAX2*, *ripN*, and *ripS5* [147]. The RipS effectors have variable impacts on the pathogenesis of bacterial wilt across different *Solanaceae* hosts, with the most pronounced effects being observed in eggplant. Notably, the genes *ripS1*, *ripS4*, and *ripS5* are crucial in the disease progression of eggplant. The *ripS*-null mutant (RK7060) exhibited attenuated proliferation and reduced virulence in eggplant [152]. In contrast, the influence of the RipS family on tomato is comparatively less significant, as the *ripS*-null mutant (RK7060) demonstrated only slightly decreased proliferation and pathogenicity compared to the wild-type strain.

It is worth mentioning that in some cases, the abilities of some T3Es are mutually antagonistic. For instance, RipAC and RipAY inhibit the HR reaction and immune responses triggered by RipE1 in *N. benthamiana* [80,115]. RipE1 is a highly conserved effector in most *R. solanacearum* strains. It triggers an HR reaction and ETI in *N. benthamiana*, the latter requiring the immune regulator SGT1. The dependence of RipE1 on SGT1 might be disrupted by the association of RipAC with SGT1, as mentioned in the previous section [80]. The suppression mediated by RipAY may rely on its ability to degrade cellular glutathione, as RipE1 perception correlates with an enhancement of cellular glutathione [115]. These studies reflect an adaptation strategy of *R. solanacearum* during its long battle against plants. In the case mentioned above, rather than losing RipE1, *R. solanacearum* has developed other effectors to suppress the induction of immunity caused by RipE1. In this context, the concept of avirulance should be considered relative to a certain effector.

#### 6.2.2. Recognition of Type III Effectors by NLRs

Among the resistance genes, the most prevalent class encodes the intracellular nucleotide-binding leucine-rich repeat receptor (NLR) resistance proteins. These NLR proteins possess a remarkable ability to either directly recognize or modulate the function of pathogen effectors during pathogen invasion. This recognition event subsequently triggers the activation of the ETI pathway, which is characterized by a rapid and robust defense response including the ROS burst, the elevation of Ca^2+^ levels, the activation of the MAPK cascade, the production of hormones, and the induction of the HR response [153,154].

NLR proteins typically possess two conserved domains, a central nucleotide-binding oligomerization domain (referred to as the NB or NOD domain) and a C-terminal leucine-rich repeat (LRR) domain. The LRR domain in NLR proteins serves as the primary site for direct or indirect interaction with pathogen effectors. The NOD domain, which harbors ATP-binding activity and is intimately associated with the oligomerization module, is essential for NLR activation [155]. NLR proteins also feature a variable N-terminal effector region such as the Toll/interleukin 1-like receptor (TIR), coiled-coil (CC) domain, or RPW8-like CC domain (RPW8). Based on these, NLRs are classified into TIR-NLR (TNL), CC-NLR (CNL), and RNL subgroups. The CC, TIR, and RPW8 domains act as signal hubs, which play a regulatory role in downstream defense reactions upon NLR activation [156]. Notably, the CC domains in specific NLRs possess a dual role to recognize effectors and interact with both the effectors and their corresponding target proteins within pathogens [157,158].

Many studies have been carried out to decipher the specific recognition patterns between NLR proteins and different types of pathogen effectors. A paradigmatic example is the *Arabidopsis* NLR pair, namely RRS1-R/RPS4, which is reported to recognize two structurally distinct effectors, AvrRps4 from *P. syringae* and the previously mentioned RipP2 from *R. solanacearum,* via the C-terminal WRKY domain in RRS1-R [94,159]. In this system, RPS4/RRS1 integrate a “decoy” domain that enables the detection of the effectors that target WRKY transcription factors [159]. Further study reveals that the recognition involves the TIR and LRR domains of the NLR proteins, as specific mutations at positions C15 in the TIR domain and L816 in the LRR domain abolished RRS1-R function and autoimmune response [160,161,162,163]. Intriguingly, a structural study showed that the β2-β3 segment of RRS1WRKY mediated the perception of AvrRps4C by RRS1 through binding an electronegative patch on the surface of AvrRps4C [164], which affects the binding activity of the WRKY domain with W-box DNA. Although the detailed mechanism underlying the interaction of RRS1-R/RPS4 and the effectors that target WRKY transcription factors have not yet been discovered, these studies present a novel mechanism by which NB-LRR receptor pairs can perceive pathogen effectors.

RESISTANCE TO RALSTONIA SOLANACEARUM RIPY (RRS-Y), a plasma membrane-localized CNL protein, has been reported to recognize the RipY and RipY-like effectors that are conserved among the diverse RSSC species. This recognition involves the phosphate-binding loop (P-loop) and cysteine residues of the CC domain within RRS-Y [165]. While the CC domain of RRS-Y was able to trigger cell death, two cysteine residues in the CC domain were essential for membrane localization of RRS-Y. A further investigation on the functional domains of RRS-Y in the future may help to decipher the broad-spectrum pathogen recognition.

The *Ptr1* gene encodes a CNL protein that was initially identified in the wild tomato *Solanum lycopersicoides* LA4245, but is a pseudogene in the cultivated tomato. Its heterogenous expression in the cultivated tomato offers resistance to bacterial wilt disease by recognizing RipBN, AvrRpt2, and RipN [166,167,168]. Although the underlying mechanism for the activation of Ptr1-mediated resistance remains largely unclear, Ptr1 appears to function in a way similar to the *Arabidopsis* resistance protein RPS2, which senses AvrRpt2 and inhibits its activity in the modification of plant Rin4 protein [169,170]. On the other hand, Ptr1 is further reported to mediate the recognition of multiple T3Es with diverse sequences from several bacterial pathogens. These effectors include AvrB, AvrRpm1, and HopZ5 from *Pseudomonas*, RipE1 from *Ralstonia*, and AvrBsT from *Xanthomonas* [171,172]. The co-expression of Ptr1 together with AvrRpm1, AvrB, HopZ5, or AvrBsT in the leaves of *N. benthamiana* [171] or with RipE1 in the leaves of *N. sylvestris* [172] resulted in a strong programmed cell death. These findings indicate that Ptr1 has the potential to serve as a broad-spectrum resistance factor against diverse bacterial pathogens.

Down-regulation of an NLR protein coding gene *Roq1* via virus-induced gene silencing (VIGS) resulted in enhanced bacterial growth and accelerated wilting in tobacco plants [173]. The resistance mediated by Roq1 might be attributed to its ability to recognize the RipB effector in *R. solanacearum* RS1000. The recognition of RipB by Roq1 has been proved to be essential for the production of ROS and the expressions of defense-related genes in *N. benthamiana* [174], which resonates with a study that found that RipB expression in a *roq1* mutant disrupted the basal defense to *R. solanacearum* in *Arabidopsis* [118].

These findings reveal the diverse functions of plant NLRs in the detection of pathogen effectors and the initiation of effective immune response. However, the precise mechanism underlying NLR-T3E recognition is still largely unknown. Further exploration of the interaction between specific T3Es and NLRs is required, which will not only deepen our understanding of the complex plant–pathogen interactions but also hold the potential to provide valuable insights in developing more effective strategies for enhancing plant resistance against a wide range of pathogens.

## 7. Conclusions and Perspectives

T3Es play a central role in the molecular dialog between *R. solanacearum* and host plants during the infection process. T3Es can interfere with host plant metabolism, gene transcription, and hormone signaling, and in turn suppress the plant’s innate immune responses. Such global influence helps to create a favorable environment for the pathogen’s colonization and proliferation, ultimately leading to the development of disease symptoms such as wilting and necrosis. However, in the evolutionary arms race, plants have also developed corresponding mechanisms to recognize T3Es and elicit a defense. The recognition involves gene-to-gene pairs comprising avirulent effectors and their corresponding receptors or resistance genes. Among them, NLR proteins are the major resistance receptors to specifically detect certain T3Es. Once recognized, they trigger a cascade of defense responses, which can limit the spread of the pathogen and confer resistance. To further explore the underlying mechanism of how T3Es enhance bacterial virulence or elicit a robust immune response in host plants, as well as to investigate the potential utilization of T3Es to boost plant resistance, several directions, including exploring the co-evolutionary relationship between T3Es and host plant resistance genes, investigating the role of T3Es in the adaptation of *R. solanacearum* to different host plants, and a more detailed characterization of the specific T3E–host molecule interactions, can be addressed in future studies. Overall, continued and intensive research on T3Es will contribute to a more comprehensive understanding of plant–pathogen interactions and facilitate the development of more effective and sustainable strategies for plant disease control.
